# Challenges in Diagnosis and Management of Atlantoaxial Tuberculosis: A Case Report

**DOI:** 10.3390/medicina61020224

**Published:** 2025-01-26

**Authors:** Chiu-Chun Chen, Chi-Ruei Li, Hsi-Kai Tsou, Ting-Hsien Kao, Ruei-Hong Lin

**Affiliations:** 1 Department of Neurosurgery, Neurological Institute, Taichung Veterans General Hospital, Taichung 40705, Taiwan; a5880018@gmail.com (C.-C.C.); fantastic1694@gmail.com (C.-R.L.); 2 Functional Neurosurgery Division, Neurological Institute, Taichung Veterans General Hospital, Taichung 40705, Taiwan; joshuakth@gmail.com (T.-H.K.); foxboxccc@hotmail.com (R.-H.L.); 3 Department of Post-Baccalaureate Medicine, College of Medicine, National Chung Hsing University, Taichung 402202, Taiwan; 4 College of Health, National Taichung University of Science and Technology, Taichung 403027, Taiwan; 5 Department of Rehabilitation, Jen-Teh Junior College of Medicine, Nursing and Management, Houlong 356006, Taiwan

**Keywords:** atlantoaxial tuberculosis, tuberculous, spondylitis, transoral approach, pyogenic, case report

## Abstract

*Background and Objectives*: Atlantoaxial tuberculosis (TB) is rare, and its diagnosis is difficult. Herein, we present a rare case with a challenging diagnostic journey of atlantoaxial TB spanning over two years. *Materials and Methods*: A 70-year-old immunocompetent female patient presented with a four-week history of nuchal pain, stiffness, and headache. She did not have any TB-associated constitutional symptoms. The result of the initial biopsy indicated only a nonfermenting Gram-negative bacillus and the histopathological report revealed concurrent acute and chronic inflammation. Posterior fusion with bilateral C1 lateral mass and C2 transpedicular screw fixation was performed after a five-week course of antibiotics. *Results*: However, the atlantoaxial abscess progressed and led to myelopathy two years later. Tuberculous spondylitis was not confirmed until the second biopsy. We chose the transoral approach for prompt abscess evacuation and to prevent unnecessary damage to the nearby vital neurovascular structures. The sputum culture and chest radiograph did not reveal concurrent pulmonary TB. *Conclusions*: Spinal TB has a greater likelihood of presenting with a cold abscess without the typical constitutional symptoms of pulmonary TB. Distinctive magnetic resonance imaging (MRI) features, such as a thin and smooth abscess wall, subligamentous spread, severe vertebral body destruction, and heterogenous vertebral wall enhancement, might help to differentiate between tuberculous and pyogenic spondylitis. We hope to offer meaningful insights to clinicians facing similar intricate scenarios, including subtle clues that may lead to a quicker diagnosis and the considerations we made while designing a treatment plan.

## 1. Introduction

Cervical osteomyelitis, an acute or chronic infection of cervical vertebrae and intervertebral, constitutes 3–11% of all cases of spinal osteomyelitis [1,2,3]. In addition to pyogenic osteomyelitis [4], tuberculous osteomyelitis caused by *Mycobacterium tuberculosis* (MTB) is also reported [5]. The prevalence of spinal tuberculosis (TB) is about 1–2% of all TB, while the cervical spine accounts for 3 to 5% of all spinal tuberculosis cases [6,7,8]. The initial presenting manifestations of cervical spine tuberculous spondylitis include fever, night sweats, weight loss, neck pain, and stiffness [9]. Because cervical spondylitis deteriorates more quickly compared to other locations of spinal spondylitis [1], early diagnosis and treatment are critical.

The diagnostic approaches for TB should be based on clinical suspicion (symptoms, contact history, prior infection, imaging), laboratory examinations, and/or histopathological findings [6,10,11]. Imaging options include plain radiographs, computed tomography (CT), and magnetic resonance imaging (MRI). CT and MRI are especially important for spinal TB, as they provide a better evaluation of bony destruction and soft tissue involvement [6,11]. Laboratory tools for diagnosing active TB include the acid-fast bacillus (AFB) smear, culture, and nucleic acid amplification (NAA) testing.

The AFB smear is a rapid method with high specificity (94~100%); however, its sensitivity can vary from approximately 50~90%, depending on the bacillary burden in the specimen and the type of microscopy used [12]. The MTB culture is the gold standard for both diagnostic confirmation and sensitivity testing of antitubercular agents [6,10,11]. It can detect as few as 10^1^ to 10^2^ bacteria per milliliter of specimen [10,11], with a sensitivity and specificity of up to 92% and 96%, respectively [13]. However, it requires 6–8 weeks [11]. NAA tests, such as the Xpert MTB/RIF Ultra assay, were recommended by the WHO in 2017 due to their short turnaround time (2~3 h), good diagnostic value, and low bacillary requirement in specimens (10^1^/mL) [11,14]. The sensitivity and specificity of pulmonary TB detection accounted for 77–70% and 86–96%, respectively [14]. The diagnostic accuracy for extrapulmonary TB varied greatly in different specimens. The sensitivity and specificity were 20–90% and 80–99%, respectively [14]. The diagnostic performance would differ in endemic and non-endemic areas. According to the local guidelines for TB diagnosis and treatment in Taiwan, physicians should consider an NAA test if TB is a probable diagnosis based on clinical suspicion, but an AFB smear presents a negative result [10].

Various therapeutic interventions are used to treat cervical spine TB, including anti-tuberculous medication and surgical procedures [6]. Pharmaceutic therapy is typically the first-line treatment. However, patients with cervical spine TB complicated with atlantoaxial instability (AAI) [7], secondary basilar invagination [15], or epidural abscesses causing spinal canal compression [8] are strongly advised to have surgery as soon as possible.

In this case report, we presented a rare case of atlantoaxial TB that initially exhibited significant symptom relief following surgical intervention but experienced dramatic neurological deterioration two years post-surgery. The challenging diagnostic journey of atlantoaxial TB, spanning over two years, is described.

## 2. Case Presentation

A 70-year-old immunocompetent female patient with a history of hypertension was admitted to the hospital, presenting with a four-week history of nuchal pain, stiffness, and headache. The patient reported no radiating pain to the upper shoulder or limbs, numbness, or focal weakness. In addition, no signs of suspected infection were reported, such as local redness, swelling, fever, pus discharge, wounds, phlegmy cough, altered states of consciousness, fatigue, weakness, or general muscle pain. There were no abnormalities found in the blood test, including the total WBC count, differential WBC counts, and platelet count, as well as the levels of hemoglobin, BUN, creatinine, sodium, potassium, and GPT.

**Figure 1:**
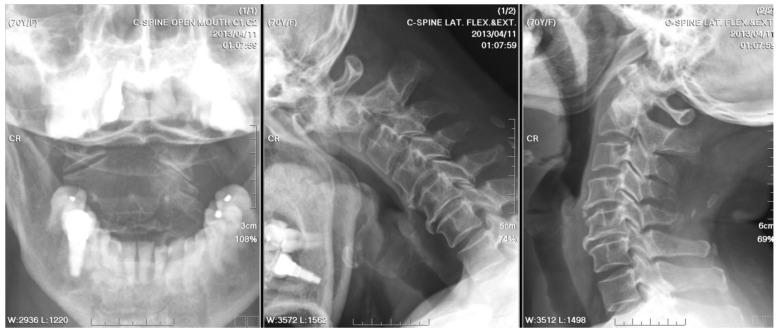
Initial radiographs of the cervical spine: They identified bony destruction of the C2 odontoid process and atlantoaxial subluxation.

**Figure 2:**
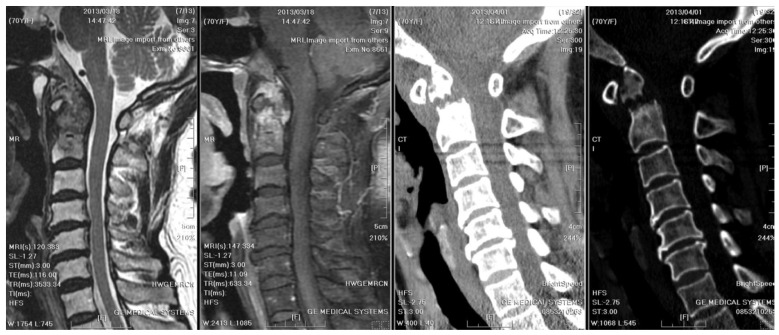
Initial MRI and CT of the cervical spine: They revealed an osteolytic lesion at C2, along with prevertebral and paravertebral soft tissue infiltration. Mild spinal canal stenosis was observed, while the diameter of the spinal cord was within an acceptable range. Bony destruction of C2 odontoid process and atlantoaxial subluxation were identified. Heterogeneous contrast enhancement of vertebral body and subligamentous spread at prevertebral area were observed. The wall of abscess was ill-defined, smooth, and regular. However, the thickness of the wall was equivocal.

A radiographic examination of the cervical spine identified bony destruction of the C2 odontoid process and C1–C2 subluxation (Figure 1). Subsequent MRI with contrast enhancement and CT revealed an osteolytic lesion at C2, along with prevertebral and paravertebral soft tissue infiltration (Figure 2). The patient was suspected to have osteomyelitis, spinal tumor, or Langerhans cell histiocytosis. Prophylactic antibiotics cefazolin and metronidazole were administrated for one day prior to surgery.

**Figure 3:**
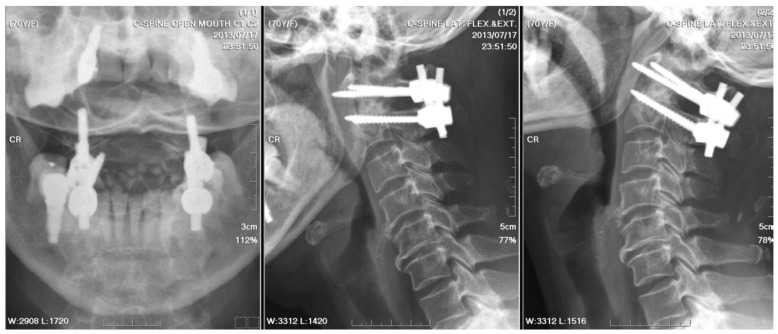
Postoperative radiographs of the cervical spine: They were checked three weeks after a five-week course of antibiotics and posterior instrumented fusion with bilateral C1 lateral mass and C2 transpedicular screw fixation. The stabilization was fair.

A C2 odontoid biopsy was performed via a transoral approach to confirm the diagnosis, revealing chronic granulomatous inflammation with acute inflammatory changes in the soft tissue. A nonfermenting Gram-negative bacillus was identified by Gram staining, but the result of the AFB test for TB was negative. Co-trimoxazole was administrated postoperatively for a week, followed by a 5-week demonstration of ampicillin/sulbactam. After posterior fusion with bilateral C1 lateral mass and C2 transpedicular screw fixation, stabilization was achieved for AAI (Figure 3). The patient recovered well after surgery, with a significant alleviation of symptoms. The patient received cervical spine plain radiographs and follow-up visits at the neurosurgery outpatient department every three months.

**Figure 4:**
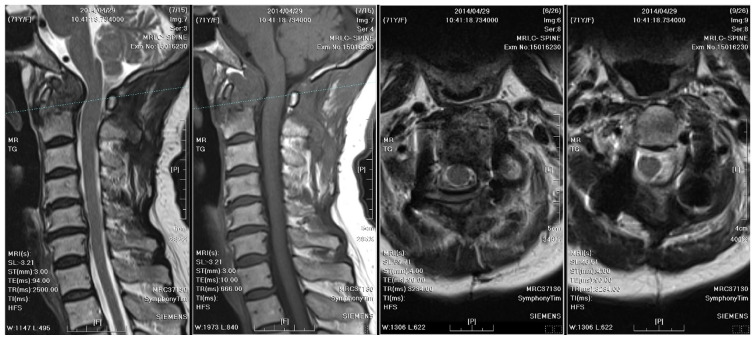
A follow-up cervical spine MRI taken 10 months after posterior surgery revealed resolution of the atlantoaxial subluxation; however, fibro-osseous hypertrophy at the atlanto-odontoid joint led to stenosis of the spinal canal.

One year after surgery, an annual follow-up cervical spine MRI revealed resolution of the C1–C2 subluxation; however, fibro-osseous hypertrophy at the atlanto-odontoid joint led to mild stenosis of the spinal canal (Figure 4).

**Figure 5:**
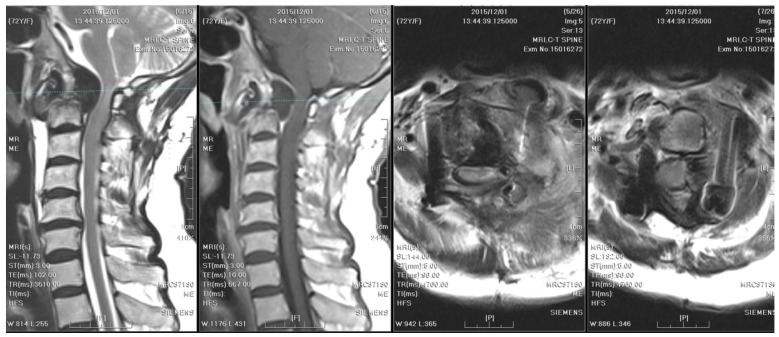
MRI of the cervical spine checked two years after the initial surgery demonstrates right rotatory atlantoaxial subluxation with bony destruction and surrounding soft tissue enhancement at the atlanto-odontoid joint, leading to severe spinal cord compression with focal myelomalacia. A thinner abscess wall (compared to the cervical spine MRI from two years ago), heterogeneously enhanced vertebral body, and subligamentous spread were observed.

Two years after surgery, the patient experienced progressive weakness in the left upper limb and numbness in both upper limbs, which dramatically worsened within a week. A follow-up cervical spine MRI with contrast showed right rotatory C1–C2 subluxation with bony destruction and surrounding soft tissue enhancement at the atlanto-odontoid joint, leading to severe spinal cord compression with focal myelomalacia (Figure 5). The atlantoaxial abscess progressed and led to myelopathy.

**Figure 6:**
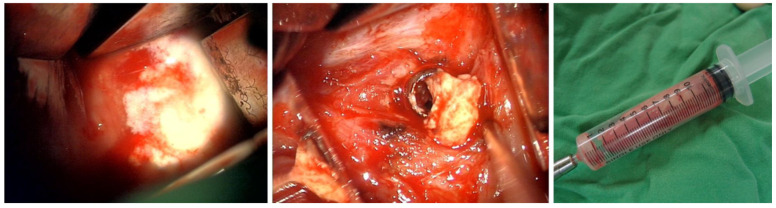
Specimen gathered via the transoral approach: Emergent decompressive surgery was performed via a transoral approach, revealing cheese-like pus and caseous necrotic tissue, with pathologic examination showing chronic inflammatory tissue, necrosis, and dystrophic calcification.

**Figure 7:**
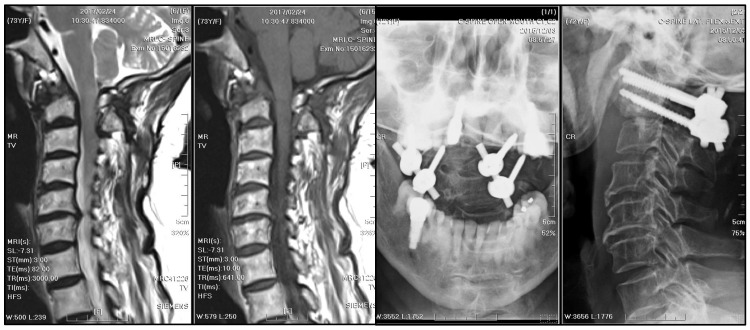
MRI and radiographs of cervical spine after antitubercular treatment: A 12-month antitubercular treatment regimen led to significant neurological improvement and a substantial reduction in the size of the mass, which became nearly undetectable on subsequent MRI. The stabilization remained fair.

Emergent decompressive surgery was performed via a transoral approach, revealing cheese-like pus and caseous necrotic tissue, with the pathologic examination showing chronic inflammatory tissue, necrosis, and dystrophic calcification. In addition, the transoral approach facilitated prompt abscess evacuation and prevention of unnecessary damage to the nearby vital neurovascular structures. The AFB smear and culture of the specimen confirmed TB spondylitis (Figure 6). However, the sputum culture and chest radiograph did not reveal concurrent pulmonary TB. Based on local guidelines for TB treatment in Taiwan [10], a 12-month antitubercular regimen was adopted. The treatment started with ethambutol/rifampin/pyrazinamide/isoniazid for three months, then shifted to rifampicin/isoniazid for nine months. During regular follow-up visits at the neurosurgery outpatient department every three months, significant neurological improvement was observed. A substantial reduction in the size of the mass, rendering it nearly undetectable, was observed on subsequent annual MRI scans (Figure 7).

## 3. Discussion

This case report describes a long and difficult process to diagnose atlantoaxial TB, which took over two years to reach a definitive diagnosis. The initial biopsy revealed only a nonfermenting Gram-negative bacillus. Histopathological analysis demonstrated a combination of acute and chronic inflammatory responses. The lack of TB exposure, prior infection, and typical TB-associated constitutional symptoms, such as fever, malaise, night sweats, significant weight loss, and anorexia, further complicated the diagnostic process. The results of the AFB smear and TB culture, which were routine investigations for osteomyelitis in our hospital, were all negative. We did not arrange a TB NAA study then due to relatively low clinical suspicion. Finally, atlantoaxial TB was diagnosed following a second transoral biopsy two years later, prompted by an abrupt onset of focal weakness. This case emphasizes the diagnostic complexities associated with atlantoaxial TB and highlights the importance of comprehensive evaluation.

Granulomatous inflammation, a hallmark of TB, can progress to caseous necrosis at the infection site and result in the formation of a cold abscess. In cases of cervical spine TB, paravertebral cold abscesses might emerge in the retropharyngeal or submandibular spaces [6]. These clinical features are much more indicative of TB than other bacterial infections. The diagnosis of spinal TB is complex and time-consuming due to the slow-growing, aerobic, and fastidious characteristics of MTB. Spinal TB, unlike pulmonary TB, often presents without constitutional symptoms, making the diagnosis more challenging [11].

### 3.1. Imaging Diagnosis of Spinal TB

Distinctive MRI characteristics help differentiate between tuberculous and pyogenic spondylitis. Tuberculous abscesses typically exhibit a thin and smooth wall, while thick, irregular, and ill-defined walls are associated with pyogenic infections [16]. TB often results in heterogeneous contrast patterns of vertebral body enhancement and bony destruction exceeding 50%, compared to pyogenic infections. On the other hand, the fragmentary or osteolytic pattern of bone lesions on CT images was a predictor for spinal TB, with an odds ratio of 3.3 [17]. Furthermore, the combination of the above CT finding and three MRI features (thin abscess wall, destruction of more than half of the vertebral body, and subligamentous spread) had an even higher diagnostic value (odds ratio of 15.58) [17]. The first cervical spine MRI and CT of our case revealed severe bony destruction (but less than 50%), heterogeneous contrast enhancement of the vertebral body, and subligamentous spread in the prevertebral area. The wall of the abscess was ill-defined, smooth, and regular. However, the thickness of the wall was equivocal (Figure 2). A cervical spine MRI performed due to progressive weakness two years later revealed a thinner abscess wall, as well as a heterogeneously enhanced vertebral body and subligamentous spread (Figure 5). From the above, although the literature has generalized several radiological traits for diagnosing spinal TB, imaging findings can still be inconclusive.

The differential diagnosis for atlantoaxial mass-like lesions encompasses a broad range of conditions, including multiple myeloma, chondrosarcoma, chordoma, metastasis, hemangioma, aneurysmal bone cyst, and retro-odontoid pseudotumors. These pseudotumors may arise from various mechanisms such as rheumatoid pannus, trauma, os odontoideum, amyloid deposition, calcium pyrophosphate deposition, Langerhans cell histiocytosis, and tenosynovial giant cell tumor [18,19,20]. Multiple myeloma is distinguished by its radiological features, including punched-out lesions, endosteal scalloping, T1 hypointensity, T2 hyperintensity, and contrast enhancement. Chondrosarcoma exhibits a characteristic “rings and arcs” appearance, T1 hypointensity, T2 hyperintensity with lobulation, and heterogeneous contrast enhancement. Chordoma, a slow-growing tumor, presents with non-specific radiological findings, such as well-defined calcified lesions on CT, T1 intermediate to low intensity, and T2 hyperintensity. Spinal metastasis, which shares many similarities with spinal TB, including vertebral destruction and disc sparing, also has non-specific radiological characteristics [21]. Hemangiomas are identified by a vertically striated, trabecular, and sclerotic pattern on the vertebral body [22], reminiscent of a corduroy fabric, with MRI findings of T1 and T2 hyperintensity and Short-TI Inversion Recovery (STIR) series hypointensity. Aneurysmal bone cysts may be noted for their internal septations, vertebral body destruction, and heterogeneous lesions with fluid–fluid levels. Retro-odontoid pseudotumors generally exhibit T1-hypointense to isointense and T2-hypointense characteristics on MRI, but specific traits to differentiate rheumatic from non-rheumatic etiologies are lacking [19,20].

### 3.2. Surgical Approaches for Cervical Spine TB

Some experts have advocated that conservative treatment may be sufficient for atlantoaxial TB. A non-surgical regimen typically consists of a medication course spanning 12–21 months, skeletal traction ranging between 3–10 days or up to 6 weeks, and the use of a rigid orthosis for several months [7,23,24,25,26]. The antitubercular regimen may consist of ethambutol/rifampin/pyrazinamide/isoniazid throughout the entire treatment course or ethambutol/rifampin/pyrazinamide/isoniazid for 2–3 months plus rifampicin/isoniazid in the maintenance phase. Behari et al. [23] hypothesized that antitubercular agents might promote fibrosis and joint stabilization.

However, current treatment recommendations advocate for surgical intervention once diffuse bony destruction and neurological deficits have developed [6]. Spinal TB frequently develops in an undiagnosed condition. As a result, once spinal TB is diagnosed, it might be severe. Surgery can lead to immediate decompression, correction, or prevention of the skeletal deformity, drainage of infection foci, and obtaining adequate specimens for confirmative diagnosis [7,11]. According to a systemic review analyzing 24 studies and 247 patients [7], the treatment failure rate (defined as the necessity of undergoing further surgical treatment) was 1.3% in the surgery group, and atlantoaxial dissociation accounted for 71.4% of treatment failures. On the contrary, the rate of poor outcomes (15%) was significantly higher in the conservative group [7]. Moreover, approximately 6% of patients under conservative treatment were complicated with fixed atlantoaxial dislocation, though they were not defined as treatment failure. Given the aforementioned reasons, surgical intervention was deemed the appropriate management for this patient.

Surgical options for atlantoaxial TB include anterior decompression, posterior instrumented fusion, and combined surgeries [27]. A shorter route to the lesions was provided via an anterior operation and thus facilitated more efficient decompression and specimen collection. Anterior decompression can be conducted via the transoral approach, the anterior retropharyngeal approach, the lateral upper cervical approach, or the traditional Smith–Robinson anterior cervical approach. The transoral approach creates the most direct pathway, but disadvantages include a deep and narrow operative field and a higher risk of mixed infections from oral floras. Moreover, there might be complications, such as tongue edema, adversely influencing speech [27]. The anterior retropharyngeal approach offers a wider operative field; not only C1 and C2 but also the lower clivus can be exposed. Therefore, contamination from the TB abscess and oropharyngeal bacteria can mostly be avoided. However, there are many vital neurovascular structures nearby, including the hypoglossal nerve, superior laryngeal nerve, mandibular branch of the facial nerve, facial artery, internal carotid artery, and internal jugular vein. The submandibular gland must also be resected intraoperatively. In general, this technique is more complex than other approaches. The lateral upper cervical approach is also commonly used. However, it is adjacent to the vertebral artery, only provides unilateral exposure to the atlantoaxial joint, and potentially requires external jugular vein ligation and carotid sheath exploration. The traditional Smith–Robinson anterior cervical approach is the route that neurosurgeons are most familiar with. It is relatively safe because there are no nearby vital neurovascular structures except the superior laryngeal nerve and hypoglossal nerve. However, the mandible is an obstacle to a wide expansion of the operative view and thorough infection foci removal. Posterior instrumented fusion offers robust stabilization. Atlantoaxial fusion alone, rather than occipital–cervical fusion, should be prioritized to preserve activity. Nevertheless, occipital–cervical fusion is indicated when there is basilar invagination or extensive atlantoaxial erosion [11].

At first, the patient in this case report complained of solely severe nuchal pain and tightness without neurological deficits. Consequently, we arranged a two-staged combined surgery for the purpose of treating a potential infection precisely prior to a posterior instrumented fusion with C1 lateral mass and C2 transpedicular screw fixation. The transoral biopsy was chosen due to a lower risk of vital neurovascular structure damage and less tissue damage. Good stability was achieved after the posterior surgery. When the abscess progressed and led to myelopathy two years later, we selected the transoral approach for prompt decompression of the spinal cord as well as specimen gathering. Second-staged occipital-cervical fusion, indicated due to right rotatory C1-C2 subluxation and extensive bony destruction, was originally planned subsequent to the completion of the antitubercular regimen. However, the patient declined the second-stage surgery because she recovered well postoperatively. Though the patient does not receive regular outpatient department follow-up visits now, she remains in contact with us and reports good functional status eight years after surgery.

## 4. Conclusions

In this case report, we present a rare case of cervical spine TB, who initially exhibited significant symptom relief following the first surgical intervention but experienced dramatic neurological deterioration two years post-surgery. We hope this case report provides some insights to clinicians encountering similar challenging scenarios to improve patient outcomes in this rare area where infectious illness and neurosurgery intersect.

## Data Availability

The data presented in this study are available on request from the corresponding author due to privacy.

## Abbreviations

AFB	Acid-fast bacillus
AAI	Atlantoaxial instability
CT	Computed tomography
MTB	Mycobacterium tuberculosis
MRI	Magnetic resonance imaging
NAA	Nucleic acid amplification
STIR	Short-TI Inversion Recovery
TB	Tuberculosis
